# Establishment of reference intervals during normal pregnancy through six months postpartum in western Kenya

**DOI:** 10.1371/journal.pone.0175546

**Published:** 2017-04-11

**Authors:** Collins Odhiambo, Paul Omolo, Boaz Oyaro, John Williamson, John Kinuthia, Daniel Matemo, Alison Drake, Grace John-Stewart, Clement Zeh

**Affiliations:** 1 Centre for Global Health Research, Kenya Medical Research Institute, Kisumu, Kenya; 2 Division of HIV/AIDS Prevention, U.S. Centers for Disease Control and Prevention, Kisumu, Kenya; 3 Department of Research Program, Kenyatta National Hospital, Nairobi, Kenya; 4 Department of Global Health, University of Washington, Seattle, Washington, United States of America; Holbæk Hospital, DENMARK

## Abstract

**Background:**

Pregnancy is associated with changes in hematological and biochemistry values, yet there are no African reference intervals for clinical management of pregnant women. We sought to 1) develop laboratory reference intervals during pregnancy and up to 24 weeks postpartum and 2) determine the proportion of women in a previous clinical trial who would be misclassified as having out-of-range values using reference intervals from a United States (U.S.) population.

**Methods and findings:**

This was a longitudinal sub-study of 120 clinically healthy, HIV-uninfected, self-selected pregnant women seeking antenatal care services at either of two public hospitals in western Kenya. Blood specimens were obtained from consented women at gestational ages 28 and 36 weeks and at 2, 6, 14 and 24 weeks postpartum. Median and 95% reference intervals were calculated for immune-hematological and biochemistry parameters and compared to reference intervals from a Kenyan and United States (U.S.) population, using Wilcoxon tests. Differences with p≤0.05 were considered significant. Some hematological parameters, including hemoglobin and neutrophils showed significant variations compared to reference intervals for non-pregnant women. Hemoglobin values were significantly lower during pregnancy but were comparable to the values in non-pregnant women by 6 weeks postpartum. CD4, CD8 and platelets were significantly elevated in early postpartum but declined gradually, reaching normal levels by 24 weeks postpartum. Using the new hemoglobin reference levels from this study to estimate prevalence of ‘out of range’ values in a prior Kisumu research cohort of pregnant/postpartum women, resulted in 0% out of range values, in contrast to 96.3% using US non-pregnant reference values

**Conclusion:**

There were substantial differences in U.S. and Kenyan values for immune-hematological parameters among pregnant/postpartum women, specifically in red blood cell parameters in late pregnancy and 2 weeks postpartum. Use of U.S. reference intervals markedly increases likelihood of out of range values, highlighting the need for suitable locally developed reference intervals.

## Introduction

With the advent of antiretroviral therapy (ART) for HIV and other interventions to improve maternal and child health, pregnant women and infants are the focus of many health programs, including prevention of mother-to-child transmission (PMTCT). Recruitment of pregnant women into clinical trials and overall clinical management require accurate laboratory reference intervals for correct interpretation and decision making [1]. Reference intervals are useful in diagnosis of health disorders, drug toxicity monitoring, disease staging, and monitoring of treatment response. Moreover, recruitment of participants into clinical studies and the interpretation of results from such studies necessitate the use of appropriate reference intervals.

While the red cell mass increases during pregnancy, the plasma volume increases more, resulting in a relative anemia. This leads to lower hemoglobin (Hb) level, hematocrit (Hct) and red blood cell (RBC) count. However, iron supplementation during pregnancy results in higher Hb levels than in non-supplemented women, indicating that iron deficient erythropoiesis plays a significant role apart from hemodilution [2]. Hb level is known to vary with gestational age, with the highest values within the first and last trimesters and lowest values during the second trimester. Similarly, Hct and RBC count decrease with increasing gestational age, while a stable higher upper reference limit for white blood cell (WBC) count during pregnancy has been reported [3, 4]. WBC count is known to peak at delivery, thus limiting the use of this parameter as a marker for infection during delivery. This increase in WBC count results primarily from an increase in neutrophil counts and a slight increase in lymphocyte counts. Serum biochemistry parameters during pregnancy and postpartum have previously been reported with mixed results [5–10]. For example, elevated serum alanine transaminase (ALT) levels was reported in one Swedish study while another showed a decrease during pregnancy [9, 11].

Currently, no data exist on reference intervals during pregnancy based on African women. Most laboratory information systems report reference values based on samples obtained from non—pregnant women, which may not be useful for clinical decisions during pregnancy. Moreover, some of the reference intervals are derived from western populations that are predominantly Caucasian, resulting in further misclassification of participants as highlighted by several African studies [12–16]. In phase I clinical trials—in absence of a control group—this is a critical issue because otherwise clinically healthy participants are misclassified as having abnormal values, hence escalating costs in terms of participant recruitment and management [14]. More importantly, there is also an increased risk of overlooking important physiologic alterations resulting from pathological conditions and of misinterpreting normal changes as pathological events [9]. This can lead to unnecessary and potentially dangerous therapeutic actions without determining the real cause of the abnormality for example, women with abnormal liver enzyme levels due to pregnancy may be treated for intrahepatic cholestasis of pregnancy or pre-eclampsia. Consequently, there is an ongoing need to update reference intervals on the most commonly used immune-hematological and biochemistry variables during pregnancy and postpartum period. We sought to 1) develop laboratory reference intervals during pregnancy and up to 6 months postpartum for clinical decision making in western Kenya and 2) determine the proportion of women in a previous clinical trial of HIV infected women (Kisumu Breastfeeding Study or KiBS) [17] who would be misclassified as having out-of-range hemoglobin values using the established reference intervals from a United States (U.S.) population.

## Materials and methods

Participants included in our analyses were recruited from a longitudinal cohort study, the Mama Salama study, the aims of which were to define the incidence of HIV infection and identify risk factors associated with HIV infection during peri/postpartum period. Study participants comprised pregnant women seeking antenatal care services at two public hospitals in the former Nyanza Province of Kenya, Bondo County hospital and Ahero sub-County hospital from May 2011 to July 2014. Women were eligible to participate if they were HIV uninfected (based on rapid HIV testing and nucleic acid amplification test [NAAT] at enrolment and subsequently negative on NAAT throughout the study), pregnant, with a plan to remain in the area until at least 9 months postpartum, willing to have serial visits at maternal child health clinic with serial HIV testing through 9 months postpartum, not on medication that could alter hematological parameters, and no clinical evidence of malaria and helminth infections. Blood specimens for hematological and biochemistry values were obtained from consented women 14 years and above at 28 and 36 weeks gestation as well as at 2, 6, 14 and 24 weeks postpartum.

### Ethical approval

The study and the consenting procedures were approved by the Kenya Medical Research Institute’s (KEMRI) ethics review committee, University of Nairobi/Kenyatta National Hospital Ethics and Research Committee (ERC) and University of Washington Institutional Review Board (IRB). Written informed consent was obtained from participants prior to any study procedure. All specimens had a unique study identification linked to the main study consent. For all critical values, participants were referred for clinical management per Kenya standard of care.

### Blood collection and HIV serology

Whole blood was collected in ethylenediaminetetraacetic acid vacutainer tubes (Becton Dickinson, Franklin Lakes, NJ) and transported at 4°C to the KEMRI HIV-research laboratory within six hours of specimen collection for processing and analysis. Initially, HIV status was determined using HIV rapid test kits as follows: Determine (Abbot Laboratories, Tokyo, Japan), and Bioline (Standard Diagnostics Inc., Korea) as a confirmatory test for any positive specimen and Unigold (Trinity Biotech Plc, Bray, Ireland), as a tie breaker. Later, HIV infection at enrolment was determined using the new Kenyan testing algorithm, with KHB (Shangai, Kehua Bioengineering Co. Ltd) as primary kit and First Response (PMC Medical Pty. Ltd) as the confirmatory kit. Unigold was used as the tie breaker for any specimen with an indeterminate result.

### Hematological analysis

Absolute WBC counts and percentages for leukocytes with differentials (neutrophils, lymphocytes, monocytes, eosinophils, and basophils), RBC with parameters Hb, Hct and mean cell volume (MCV), and platelet counts were determined from whole blood using a Coulter ACT 5Diff CP analyzer (Beckman Coulter, France). This was performed within 24 hours of specimen collection as recommended by the manufacturer (www.beckmancoulter.com).

### Biochemistry analysis

Clinical chemistries were analyzed from serum obtained from serum separation tubes (Becton Dickinson, Franklin Lakes, NJ). Specimens were analyzed for ALT, aspartate aminotransferase (AST), bilirubin (Bil) and creatinine (Cr) using the Cobas Integra 400 plus biochemistry analyzer (Roche, Germany) per the manufacturer’s instructions (www.usdiagnostics.roche.com).

### Quality control

Quality control protocols included running known standards each day before testing specimens. In addition, the laboratory is enrolled in external quality assurance testing programs with the College of American Pathologists (CAP) (hematology, and clinical chemistry) and the United Kingdom National External Quality Assurance Service (UK NEQAS) (lymphocyte immunophenotyping and hematology). The laboratory has satisfactory performance in UK NEQAS (Lymphocyte Immunophenotyping) and CAP Clinical Chemistry as well as CAP Hematology over the past three years.

### Statistical analysis

Based on the CLSI guidelines recommendation of 120 reference subjects for establishing reference intervals and assuming a loss to follow up rate of 20%, 150 pregnant women were targeted for enrollment. Every 6^th^ woman in the parent study was targeted for enrollment into the reference interval sub-study. All data available at each time point were entered into an Access database and the median and 95% reference intervals (2.5 and 97.5 percentiles) for immune-hematological and biochemistry parameters calculated using SAS (SAS system for Windows 9.2; SAS, Inc., Cary, NC). These values were compared to established reference intervals developed for non-pregnant women in western Kenya [16], using the Wilcoxon test. Differences with p≤0.05 were considered significant.

In order to demonstrate the use of appropriate reference intervals for a given population, we determined how many of 522 participants in a previous clinical trial, KiBS [17], would have out-of-range values using the newly established reference intervals in the current study, the intervals for non-pregnant women in western Kenya and the intervals from a U.S. population of non-pregnant women [18]. Additionally, we determined the number of women who would have abnormal values using the 2004 National Institutes of Health Division of AIDS (DAIDS) toxicity tables [19].

## Results

**Fig 1:**
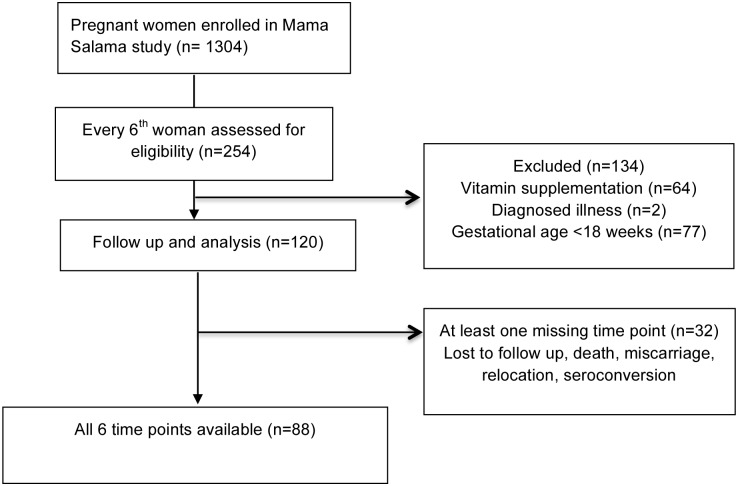
Flow diagram of participants in the study. The sub study assessed every 6th participant in the parent study for eligibility (254 participants). One hundred and twenty paticipants were enrolled and followed up to 24 weeks postpartum. Of 120 enrolled participants, 32 did not complete all six study visit measurements due to a number of reasons including participant unable to be contacted, miscarriage, death, relocation and seroconversion.

Overall, 120 women from the intended 150 were enrolled into this study because the parent study ended before achieving its intended sample size of 2000 pregnant women. Age ranged from 14 to 44 years with a median of 22 years. Of 120 pregnant women enrolled, 32 did not complete all six study visit measurements (Fig 1).

Their hematologic and biochemistry values were included in the analysis at each time point available. The 2.5 and 97.5 percentiles for each time period are presented in Table 1.

**Table 1:** Reference intervals for immune-hematological and biochemistry parameters for HIV uninfected women during pregnancy and postpartum compared to non-pregnant women. Kenya and United States, 2012–2015.

	Non-Pregnant women		Pregnant/postpartum women from this study					
Parameter	Kenya [16]	US [18]	Gestation period		Postpartum period			
Number of participants	140	NA	**120**	**92**	**88**	**95**	**101**	**99**
			**28 wk**	**36 wk**	**2 wk**	**6 wk**	**14 wk**	**24 wk**
**ALT (U/L)**	0–61	0–35	0–26	0–37	0–34	0–40	0–41	0–67
**AST (U/L)**	0–50	0–35	0–39	0–44	0–41	0–39	0–45	0–46
**Bilirubin (μmol/L)**	5.1–40.7	5.1–17.0	1.4–26.3	2.2–22.1	1.9–18.6	2.1–17.0	2.3–19.1	1.6–22.0
**Cr (μmol/L)**	0–113	0–133	0–59	0–67	0–74	0–72	0–75	0–78
**Abs CD4**	444–1488	404–1612	419–1567	466–1504	630–1741*	641–1720*	578–1880*	483–2086
**Abs CD8**	211–1078	220–1129	156–1049	195–1765	396–1458*	360–1342*	356–1267*	302–1442
**Hct (%)**	23.2–44.2	36.0–46.0	21.4–38.1*	17.7–38.6*	21.0–44.4*	26.9–43.7	27.0–44.7	27.4–43.7
**Hb (g/dL)**	8.0–14.2	12.0–16.0	6.1–13.0*	5.5–12.7*	6.5–14.7*	7.8–14.4	7.9–14.7	8.0–14.6
**MCV (fL)**	60–93	80–100	58–101	59–99	59–93	59–95	60–92	62–97
**RBC (x10**^**12**^**/L)**	3.3–5.6	4.0–5.2	2.8–4.8*	2.8–4.9*	2.9–5.8*	3.6–5.7	3.5–5.7	3.7–5.6
**WBC (x10^9/L)**	3.3–9.3	4.5–11.0	3.6–11.1	3.3–10.7	3.6–8.9	3.2–9.6	2.8–7.9	2.9–10.3
**Neut (x10^9)**	0.9–5.2	1.8–7.7	1.9–7.1*	2.0–5.7*	1.1–5.5	1.0–5.5	1.0–3.5	1.0–4.4
**Monocytes**	0.2–0.8	0–0.1	0.1–0.9	0.1–0.8	0.1–0.6	0.1–0.5	0.1–0.5	0.1–0.7
**Lymphocyte count**	1.1–3.5	0.2–0.4	0.9–3.3	1.2–3.8	1.3–3.9	1.5–3.8	1.4–3.6	1.1–4.1
**Basophils**	0.02–0.18	0–0.03	0.01–0.12	0.01–0.09	0.02–0.12	0.02–0.16	0.02–0.12	0.02–0.13
**Eosinophils (x10**^**9**^**/L)**	0–1.7	0–0.1	0–0.9	0–1.0	0–1.2	0–1.8	0–1.2	0–1.4
**Platelets (x10**^3^**/μL)**	103–390	150–350	98–395	105–425	180–607*	120–443	119–438	133–437

*p≤0.05 for comparison with non-pregnant reference interval for Kenya

^[16]^From non-pregnant cohort in Western Kenya (Zeh et al, 2011)

^[18]^From non-pregnant United States (U.S.) population (Kratz et al, 2004)

Definitions: ALT: alanine transaminase, AST: aspartate transaminase, Bil: bilirubin, Cr: creatinine, Abs: absolute, Hct: hematocrit, Hb: hemoglobin, MCV: mean corpuscular volume, RBC: red blood cells, WBC: white blood cells, NA: Not available, Neut: neutrophils, Eosn: eosinophils, Plt: platelets, fl: femtolitres, U/L: microns per litre, g/dl: grams per decilitre.

The serum biochemistry parameters were similar to those of non-pregnant women regardless of pregnancy status (Table 1). Serum biochemistry parameters, ALT, AST, Cr and Bil generally remained unchanged throughout pregnancy and postpartum and were within the reference intervals limits for non-pregnant women (Table 1). Table 2 summarizes the number of women in KiBS who would have been considered as having out-of-range hemoglobin values when reference intervals for pregnancy/postpartum is compared to the locally established reference intervals and reference intervals from the U.S. population for non-pregnant women. Overall, 0% of women in KiBS would have out of range values using this study’s pregnant cohort’s reference ranges, 55 (10.6%) of women in KiBS would be out of range compared to local non-pregnant reference ranges, and 499 (96.3%) women in KiBS had at least one out-of-range hemoglobin value using US reference intervals for non-pregnant women. Using the locally established reference intervals for non-pregnant women rather than those specific to pregnancy/postpartum women, at least more than 8% of our study population would have out-of-range Hb values during late pregnancy. Using the U.S. non-pregnant population reference intervals, over 96% of pregnant women at 28 or 36 week gestation would have been considered as having out-of-range values. Furthermore, using reference data from the 2004 DAIDS toxicity tables, we observed that among pregnant women at either 28 or 36 weeks gestation, over 76% would have been considered to have any grade of anemia, and over 25% would have been considered to have anemia grade 3 or above.

**Table 2:** Comparison of out-of-range values and frequency of adverse events among HIV infected pregnant/postpartum women in the Kisumu Breastfeeding Study (KiBS) obtained from using locally-established reference intervals for pregnant and non-pregnant women in Kenya, United States reference intervals and DAIDS toxicity tables. 2012–2015.

	Proportion of KiBS cohort out of range using reference interval for pregnancy/postpartum HIV uninfected women (this study MSS cohort)		Proportion of KiBS cohort out of range using reference Kenya reference intervals for non-pregnant HIV uninfected women [16]		Proportion of KiBS cohort out of range using reference U.S. reference intervals for non-pregnant HIV uninfected women[18]		Proportion of KiBS cohort out of range using reference 2004 DAIDS Toxicity Table [19]			
Time point							Any Grade		Grade 3&4	
	n	%	n	%	n	%	n	%	n	%
**28wk**	**0**	**0**	**55**	**10.6**	**499**	**96.3**	**397**	**76.6**	**141**	**27.2**
**36 wk**	**0**	**0**	**41**	**8.4**	**469**	**96.1**	**376**	**77.0**	**124**	**25.4**
**2 wk pp**	**9**	**1.9**	**31**	**6.6**	**309**	**65.7**	**222**	**47.2**	**69**	**14.7**
**6 wk pp**	**10**	**2.2**	**11**	**2.4**	**309**	**67.6**	**180**	**39.4**	**26**	**5.7**
**14 wk pp**	**7**	**1.6**	**7**	**1.6**	**266**	**50.4**	**115**	**25.7**	**15**	**3.3**
**24 wk pp**	**5**	**1.1**	**5**	**1.1**	**205**	**47.1**	**84**	**19.3**	**11**	**2.5**

^16^From non-pregnant cohort in Western Kenya (Zeh et al, 2011)

^18^From non-pregnant United States (U.S.) population (Kratz et al, 2004)

^19^From 2004 NIH DAIDS toxicity tables (DAIDS, 2004)

Definitions: US: United States, DAIDS: Division of AIDS, pp: postpartum

Using the reference intervals for pregnancy and postpartum from our study, up to 2.2% of KiBS participants would have been classified as having out-of-range hemoglobin values at any time point during the postpartum period. Using locally established reference intervals for non-pregnant women, up to 6.6% of KiBS participants would have been classified as having hemoglobin values out of range at two weeks postpartum, after which there was no difference between reference intervals established in the current study and those established for non-pregnant women. Using reference intervals from a U.S. population, KiBS participants with out-of-range hemoglobin values decreased over the postpartum period from 65.7% at 2 weeks to 47.1% at 24 weeks postpartum. Similarly, using the DAIDS toxicity tables, the proportion of KiBS participants with any grade anemia postpartum decreased from 47.2% 19.3%, and those with grade 3 and above anemia postpartum decreased from 14.7% to 2.5%.

The upper limit of reference intervals for WBC count was elevated in late pregnancy but was similar to the levels in non-pregnant women by 6 weeks postpartum. The lower limit of absolute neutrophil counts was elevated during late pregnancy but was similar to the levels in non-pregnant women postpartum. Similarly, the lower limits of CD4 and CD8 cells were elevated postpartum and remained as such but resolved by 24 weeks postpartum. Other WBC differentials remained relatively unchanged during pregnancy and postpartum, and most parameters were within the reference limits for non-pregnant women (Table 1).

## Discussion

Laboratory tests are often requested during pregnancy to exclude pathological complications that may affect maternal or fetal health. Diagnostic accuracy is based on evaluation of results in relation to reference values of the local laboratory. Although changes in normal laboratory values induced by pregnancy are well known, very few studies have been conducted to establish reference intervals for pregnant women. In this study, we report the 2.5th and 97.5th percentiles reference intervals for selected hematologic and biochemistry laboratory parameters in late pregnancy and up to 24 weeks postpartum. Unlike other African studies [1, 20], this was a longitudinal study that followed pregnant women from the second trimester of pregnancy to 24 weeks postpartum. Establishment of suitable reference intervals for pregnant and postpartum women has the potential to improve diagnostic quality, which could lead to increased survival, reducing unnecessary treatment and cost savings.

Hemoglobin and other red blood cell indicators were most affected during pregnancy, consistent with findings from European studies [2, 21]. This is due to normal expansion of plasma volume during pregnancy with a concomitant lower expansion in red-cell volume [22]. However, just as observed for African reference values for non-pregnant women, our values were significantly lower than European intervals for pregnant women [2, 21], reinforcing the need to have locally established reference intervals for this population. The lower limit for Hb during pregnancy was significantly lower among Kenyan pregnant women in this study compared to Caucasian women [2, 23]. This may be due to a genetic factor as indicated by lower values for non-Caucasian pregnant women compared to Caucasian women within the same environment [23]. However, values for African pregnant women is much lower than those of non-Caucasians indicating that the lower levels observed may be exacerbated by other factors including malnutrition, malaria or sickle cell disease which are endemic in this region [13, 16]. The MCV was unaffected by pregnancy and remained the same as the levels in non-pregnant women, indicating that other red blood indices were affected by hemadilution rather than nutritional deficiencies.

The upper limit of WBC count reference interval was elevated during pregnancy but was similar to the levels in non-pregnant women by 6 weeks postpartum. This may have been due to the significant increase in neutrophil count during pregnancy, possibly related to stress response, redistribution of WBCs between the marginal and circulating pools or pain, nausea, vomiting, and anxiety in the absence of infection [24]. Other WBC differentials remained relatively unchanged during pregnancy and postpartum and most parameters were within the reference limits for non-pregnant women. Reference intervals for WBC and neutrophils in the current study were generally lower than the levels in pregnancy among European women. This may be attributed to genetic, environmental or dietary factors [25, 26]. Similar to or study, there was significant elevation of neutrophil counts during late pregnancy which resolved after delivery [8].

Serum biochemistry parameters, ALT, AST, Cr and Bil generally remained unchanged throughout pregnancy and postpartum and were within the reference intervals limits for non-pregnant women. Likewise, serum biochemistry reference intervals were similar to those of European pregnant women [8] except Bil which had a higher upper limit among pregnant women in the current study. This may result from RBC hemolysis due to malaria or sickle cell disease [16]. CD4 and CD8 levels were elevated postpartum compared to the levels in pregnancy but by 24 weeks postpartum gradually approached the levels in non-pregnant women. This unexpected observation has previously not been reported and this difference may be due to the longitudinal design of this study where the same women are followed from pregnancy to postpartum compared to majority of studies, which are cross-sectional.

While it is possible that the observed differences between African and Caucasian populations may be due to differences in pre-analytical factors that may differ from the US and European settings, we would attribute these differences to environmental and genetic factors for two reasons. Firstly, many studies in the African setting have reported significantly lower hematologic parameters compared to values obtained from Caucasians [12–16, 27]. Secondly, within the US setting, lower values have been observed for participants of African descent [23]. Moreover, our group has conducted two studies, one in which specimen were obtained in the field and transported to the testing laboratory and a second study in which specimen were drawn from the laboratory setting with no significant differences observed for hematologic parameters [16, 27]. While parasitic infections like helminth and malaria infections are endemic within the study region, there were no women with evidence of the former and only 2 cases of the later probably as a result of malaria prophylaxis provided as part of antenatal care.

A limitation of this study is that it did not include a concurrent sample of non-pregnant women from the same reference population. However, we recently demonstrated that the reference intervals established for western Kenya [16] are valid for use for a different population sampled from the same region [24]. Another limitation is that we were unable to recruit women early enough to capture the first trimester of pregnancy due to the design of the parent study. Nevertheless, our data represent the first reference intervals developed in Kenya for potential use locally in the clinical management of women during pregnancy and postpartum periods.

In conclusion, there were substantial differences in U.S., European and Kenyan reference values for immune and hematological parameters among pregnant and postpartum women, specifically in red blood cell parameters including hemoglobin in late pregnancy and 2 weeks postpartum. Using U.S. reference intervals for hemoglobin would result in an increase in out-of-range values among pregnant/postpartum women participating in a previous clinical trial highlighting the suitability of locally developed reference intervals. In the present study, we have established reference intervals for a number of hematological and biochemistry variables in healthy pregnant women that can be used for clinical management and recruitment into clinical trials in western Kenya. These values should be considered for development of region specific reference intervals and toxicity tables for pregnancy/postpartum women in Africa.
